# Non-Isothermal Crystallization Behavior and Thermal Properties of Polyethylene Tuned by Polypropylene and Reinforced with Reduced Graphene Oxide

**DOI:** 10.3390/nano10081428

**Published:** 2020-07-22

**Authors:** Antimo Graziano, Otavio Augusto Titton Dias, Christian Garcia, Shaffiq Jaffer, Jimi Tjong, Mohini Sain

**Affiliations:** 1 Centre for Biocomposites and Biomaterials Processing, University of Toronto, 33 Willcocks Street, Toronto, ON M5S 3BS, Canada; antimo.graziano@mail.utoronto.ca (A.G.); otavio.dias@mail.utoronto.ca (O.A.T.D.); jtjong@ford.com (J.T.); 2 Bahen Centre for Information Technology, University of Toronto, 40 St. George Street, Toronto, ON M5S 2E4, Canada; christian.garciasalguero@mail.utoronto.ca; 3 TOTAL American Services Inc., 82 South Street, Hopkinton, MA 01748, USA; shaffiq.jaffer@total.ca; 4 Department of Mechanical and Industrial Engineering, University of Toronto, 5 King’s College Road, Toronto, ON M5S 3G8, Canada

**Keywords:** Reduced Graphene Oxide (RGO), Thermal Gravimetric Analysis (TGA), Heat Deformation Temperature (HDT), Differential Scanning Calorimetry (DSC), non-isothermal crystallization kinetics

## Abstract

This research work is the first to report thermal stability, heat deformation resistance, and crystallization behavior of a Polyethylene (PE)-based biphasic polyolefin system reinforced with Reduced Graphene Oxide (RGO), which was obtained through Graphene Oxide (GO) chemical reduction. Polypropylene (PP) represented the polymeric dispersed phase. A strategic PE/PP/RGO manufacturing procedure was employed to thermodynamically localize RGO at the PE/PP interface, as confirmed by Transmission Electron Microscopy (TEM), bringing a uniform micro phase dispersion into the macro phase. In addition, studies of PE non-isothermal crystallization kinetics indicated that the morphology tunable micro phase and the nanolayered RGO promoted a nucleation-controlled PE crystallization, which was supported by Polarized Light Optical Microscopy (PLOM). This, together with fine morphology, justified the remarkable enhancement registered for the ternary system’s thermal stability and heat deformation resistance. Different filler loads were employed, with weight fractions of 2% and 4%. It was observed that the former, being better exfoliated and more homogeneously distributed at the PE/PP interface than the latter, led to a more improved PE crystallization, alongside a greater ternary system’s thermal properties. Moreover, the thermal stability of PE/PP reinforced with 2% of RGO was even higher than that of virgin PP, while their heat deformation resistance values were found to be similar. Therefore, this unique outcome provides industries, such as the energy and automotive sectors, with the opportunity to substitute PP-rich products with those mostly comprised of a cheaper, more abundant, yet performant PE.

## 1. Introduction

Over decades, polymers have been replacing aluminum, metal, and steel in many industrial applications, because they are lightweight, reduce manufacturing expenses, and have improved thermal and mechanical properties [1]. More recently, polymer blending has become a common strategy for producing a novel material that combines the good properties of the two homopolymers, while saving cost, making the unique product suitable for highly demanding applications [2]. Other advantages of polymer blending include faster and more flexible manufacturing compared to having to synthesize the final product from scratch, and most importantly, producing sustainable products by recycling plastic waste [3]. The binary system comprised of Polyethylene (PE) and Polypropylene (PP) is gaining a great deal of attention, because both polymers are very abundant and have good processability [4]. Usually, PP has better properties than PE, while the latter is cheaper. Thus, industries are interested in developing a PE/PP binary system, with PE being the major phase. This way, the small amount of PP will not only save cost but will also transfer its good properties to the PE matrix, allowing the manufacturing of a high-performance and cost-effective PE dominant material, which could eventually replace pure PP in many industrial applications. Nevertheless, a positive Gibbs Free Energy for the PE/PP binary system translates into thermodynamic immiscibility between the two polymers. This results in weak interfacial adhesion and hence poor blend properties [5]. A lot of research has been conducted on PE/PP compatibilization by employing a co-polymer as the third component [6], through non-reactive [7] and reactive compatibilization [8]. Nevertheless, targeted fillers, which are solid additives such as fibers, spheres, platelets, etc., have recently been seriously considered as compatibilizers because of their superior properties compared to common copolymers. As a result, this unique co-factor can significantly increase the compatibility between the two polymers and the material’s performance [9]. When the employed reinforcing agent is nanosized, its high aspect ratio promotes a very fine dispersion in the binary system, along with a high surface area of interaction with the two polymers. Therefore, nanofillers [10] are replacing conventional fillers [11] in the reinforcement of PE/PP binary systems. However, these novel co-factors are expensive; thus, the mass production of biphasic polyolefin systems reinforced with targeted fillers is very limited [12]. Among all nanofillers, graphene can be synthesized through flexible procedures, such as Graphene Oxide (GO) modification, in a very cheap way [13]. Additionally, graphene has remarkable structural and performance attributes [14]; thus, it can greatly enhance the overall properties of biphasic polyolefin systems, while minimizing cost. One of the most employed GO modification procedures is reduction with hydrazine, leading to Reduced Graphene Oxide (RGO). On the other hand, very few studies have been conducted on the impact of RGO on PE/PP compatibilization and performance enhancement. Tu and coworkers studied how RGO affects PE/PP electrical and mechanical properties, when employing different mixing times and speeds [15], as well as different orders of mixing [16]. However, the literature is lacking reports on PE/PP/RGO thermal stability and heat deformation resistance. In addition, studies of PE crystallization behavior, in the ternary system PE/PP/RGO, are absent. Therefore, the aim of this work is to give a novel and unique contribution to the research by investigating, for the first time, PE/PP/RGO thermal properties such as thermal stability and heat deformation resistance. Estimation of thermal degradation activation energy is also presented, using the Kissinger method [17], for quantifying PE/PP/RGO thermal stability. In addition, this research work reports the first study of PE non-isothermal crystallization kinetics (paired with Polarized Light Optical Microscopy (PLOM) studies), in the ternary system PE/PP/RGO, following the method developed by Liu [18]. Herein, the investigation of isothermal crystallization kinetics is not outlined, since industrial processes are generally conducted under dynamic non-isothermal conditions. Lastly, the thermal stability of the PE-rich material presented here exceeds that of virgin PP, whereas their heat deformation resistance values are similar. Therefore, the key findings of this study open opportunities for industrial sectors, such as energy and automotive, to substitute PP-rich products with those mainly based on a more abundant, cheaper, yet performant PE. This will bring a remarkable progress in the engineering of advanced and lightweight polyolefin-based materials that are suitable for today’s high-demanding applications.

## 2. Materials and Methods

### 2.1. Materials

Isotactic Polypropylene (iPP), with the trade name PPH 3060, was purchased from Total SA, La Porte, TX, USA. The Melt Flow Index (MFI), density, and melting temperature (T_m_) were 1.8 g/10 min, 0.905 g/cm^3^, and 165 °C, respectively. Total SA, La Porte, TX, USA, also supplied High-Density Polyethylene (HDPE), with the trade name BDM2-15/10. Its MFI is 1.2 g/10 min, its density 0.956 g/cm^3^, and its T_m_ 134 °C. Sigma-Aldrich, Oakville, ON, Canada, was the supplier of GO, hydrazine monohydrate, and methanol.

### 2.2. Preparation of RGO

A fine dispersion of 1.5 g of as-received GO in 1.5 L of deionized water was achieved by sonication, for 2 h. Afterwards, hydrazine monohydrate (15 mL) was introduced, reacting with GO at 95 °C, for 5 h, under vigorous stirring. Next, washing and filtration were performed with an excess of deionized water, followed by drying at room temperature for 24 h. Lastly, the sample was vacuum dried at 80 °C for 24 h.

### 2.3. Preparation of RGO Filled HDPE/iPP Biphasic Systems

The synthesis of HDPE/iPP/RGO was performed in two steps. The first one was solution mixing of 0.85 g of RGO with 4.15 g of iPP. In detail, the components were added to a flask with 300 mL of xylenes and mixed for 30 min at 140 °C, under stirring and nitrogen atmosphere. Then, precipitation of the hot content of the flask was performed in 300 mL of methanol, followed by filtration. Afterwards, washing with plenty of deionized water was performed to remove most of the xylenes/methanol solution. Following this, the sample was first dried at room temperature for 24 h and then vacuum dried at 80 °C for 24 h to completely remove any remaining trace of solvent. The second step was melt mixing, in a Brabender batch mixer, 7 g of the masterbatch iPP/RGO with 53 g of HDPE. However, in order to achieve a better dispersion of iPP/RGO into HDPE, the latter was first fully melted at 180 °C for 3 min at a rotor speed of 80 rpm, and only afterwards, iPP/RGO was introduced. At this point, the speed was increased to 120 rpm, and the mixing was performed for 2 min at 180 °C. During this two-step manufacturing procedure, the co-factor was thermodynamically driven to self-localize at the biphasic polyolefin system interface, as explained in detail in Section 3.1. A 90/10 weight ratio was chosen for HDPE/iPP, and the weight fraction of RGO was 2%. One of the objectives of this research work was to investigate the impact of filler amount on the material’s properties. Thus, biphasic polyolefin systems with 4 wt % of RGO were also produced. In addition, for comparison purposes, unfilled binary systems, as well as those filled with GO, were synthesized as well. Lastly, for simplicity, sample denotations were generated (Table 1).

### 2.4. Thermal Gravimetric Analysis (TGA)

TGA was carried out with a Thermal Gravimetric Analyzer, TA instruments TGA Q500, Pittsburgh, PA, USA. Each sample underwent degradation from room temperature to 600 °C, in nitrogen atmosphere, at four different heating rates (10, 20, 30, and 40 °C/min). The material’s thermal stability was quantified by estimating the activation energy of thermal degradation (*E_T_*), following the Kissinger method: [19].

(1) $$\ln\;\left(\frac{\beta_{0}}{T_{max}^{2}}\right)=\ln\left(\frac{A\times R}{E_{T}}\right)-\frac{E_{T}}{R\times T_{max}}.$$

*R* is the gas constant, *T_max_* is the temperature of maximum degradation, *β*_0_ is the cooling rate, and A is the pre-exponential factor. *E_T_* could be calculated from the slope of the plot “ln(*β*_0_/*T_max_*^2^)” versus “1/*T_max_*”. Tests were redone two more times to incorporate the standard deviation values.

### 2.5. Heat Deformation Temperature (HDT) Characterization

HDT was measured employing a Q800 DMA analyzer (TA Instruments, Pittsburgh, PA, USA). The testing method was three-point bending, and the heating rate was 2 °C/min, from room temperature to 70 °C. Each sample had a length of 50 mm (*L_DMA_*), a width of 5.5 mm (*W_DMA_*), and a thickness of 0.45 mm (*T_DMA_*). By following ASTM D648, a 0.455 MPa stress (σ) was applied, and the Dynamic Mechanical Analysis (DMA) analyzer was set to show the plot “sample strain” (or sample deflection) versus “temperature”. The strain (or deflection) at which the deflection temperature was taken could be calculated through the following procedure. Given the applied stress (0.455 MPa) and the sample dimensions, Equation (2) allowed the calculation of the applied force (*F*):

(2) $$F=\frac{2}{3}\left[\sigma \left(\frac{T_{DMA}^{2}\times W_{DMA}}{L_{DMA}}\right)\right].$$

In our case, *F* = 6.8 kN. Next, the strain (*ε*) in the American Society for Testing and Materials (ASTM) sample, at a deflection (*d_ASTM_*) of 0.25 mm, could be calculated:

(3) $$\varepsilon =6\left(\frac{d_{ASTM}\times T_{ASTM}}{L_{ASTM}^{2}}\right)=0.00121=0.121\%.$$

*T_ASTM_* = 13 mm and *L_ASTM_* = 127 mm are the sample thickness and length, respectively, according to ASTM D648. Lastly, the sample deflection (*d_DMA_*) at which the deflection temperature was taken could be calculated:

(4) $$d_{DMA}=\varepsilon \left[\frac{L_{DMA}^{2}}{\left(6\times T_{DMA}\right)}\right].$$

In our case, *d_DMA_* = 1.8 mm. Four specimens were considered for each test.

### 2.6. Differential Scanning Calorimetry (DSC)

DSC tests were performed using a Q1000 Differential Scanning Calorimeter, from TA Instruments, Pittsburgh, PA, USA. In nitrogen atmosphere, each sample was first heated from room temperature to 180 °C to erase thermal history and then cooled down to room temperature, which was followed by reheating up to 180 °C. The heating and cooling cycles were performed at a rate of 10 °C/min. Tests were redone two more times to incorporate the standard deviation values. Crystallization temperature (*T_c_*), melting point (*T_m_*), and percentage crystallinity (*X_c_*) of PE in the samples PE90, PE90GO2, PE90RGO2, PE90GO4, and PE90RGO4 were obtained from cooling thermogram, heating thermogram, and Equation (5), respectively:

(5) $$X_{c}=\frac{\Delta H}{\;w_{PE}\times \Delta H_{0}}\times 100.$$

Δ*H*_0_ indicates the melting enthalpy of PE for 100% crystallinity, and its value is 293 J/g [20]. Conversely, the melting enthalpy of PE is represented by ΔH. Moreover, the data were normalized to account for the content of micro phase (PP) and filler (GO or RGO). Indeed, *w_PE_* is the PE weight fraction in each sample. Studies of PE non-isothermal crystallization kinetics in unfilled and filled binary systems were performed by running DSC tests at cooling rates of 10, 15, 20, and 25 °C/min. The Kissinger method [21] allowed the calculation of the activation energy of crystallization (*E_c_*) of PE, in the different samples investigated, by considering the variation of crystallization temperature as a function of the cooling rate:

(6) $$\frac{\left[ln\left(\frac{\beta_{0}}{T_{c}^{2}}\right)\right]}{\frac{1}{T_{c}}}=\frac{-E_{c}}{R}.$$

The crystallization temperature of PE is represented by *T_c_*. *R* indicates the gas constant, while *β*_0_ represents the cooling rate. The slope of the plot “ln(*β*_0_/*T_c_*^2^)” versus “1/*T_c_*” gave the value of *E_c_*. Tests were redone two more times to incorporate the standard deviation values.

The Liu method [18] was employed to obtain another PE crystallization parameter, *F*(*T*), which is related to the cooling rate needed to obtain a certain crystallinity degree, at unit crystallization time:

(7) $$\log\beta_{0}=\log F\left(T\right)-b\log t_{c}.$$

The Avrami/Ozawa exponents ratio is indicated by b = n/m, while *β*_0_ represents the cooling rate, and *t*_c_ represents the crystallization time. Equation (8) allowed the calculation of the crystallization time:

(8) $$t_{c}=\frac{T_{0}-T_{c}}{\beta_{0}}.$$

The temperature at which crystallization starts is indicated by *T*_0_. The intercept with the x axes of the “ln(*β*_0_)” versus “ln(*t_c_*)” plot generated the value of *F*(*T*). Tests were redone two more times to incorporate the standard deviation values. The Khanna method [22] was employed to estimate the PE “crystallization rate coefficient” (CRC), which is indicative of the rate of the non-isothermal crystallization:

(9) $$CRC=\frac{\Delta \beta_{0}}{\Delta T_{c}}.$$

The *CRC* value was obtained from the slope of the “*β*_0_” versus “*T_c_*” plot. Tests were redone two more times to incorporate the standard deviation values.

### 2.7. TEM

The exfoliation state of GO and RGO, as well as its localization (inside the PE phase, inside the PP phase, or at the PE/PP interface) were investigated with a Transmission Electron Microscope, FEI Tecnai 20, Wilmington, NC, USA. A diamond knife was used to cryogenically cut each sample at −140 °C, obtaining ultrathin films. Then, these were placed onto carbon-coated Cu grids, with standard 400 mesh. TEM images were taken at an accelerating voltage of 200 kV.

### 2.8. PLOM

A Polarized Light Microscope, Olympus BX50 (Spectra Services, Ontario, NY, USA), was employed to observe the PE crystal growth and size distribution. Each specimen (50 µm thick) was embedded between two glass cover slips and then heated up to 180 °C by using a hot stage, Linkam TMS 94 (Linkam Scientific Instruments, Tadworth, UK). This temperature was held for 3 min, ensuring the complete melting of the sample, hence the removal of thermal history. Afterwards, a quick cooling to 115 °C was performed, and this temperature was held to observe isothermal crystal growth. Images were taken after 5 min by using a Charged Coupled Device (CCD) camera, Donpisha XC-003 (Spectra Services, Ontario, NY, USA). The spherulite average diameter (*d_avg_*) was measured with the software ImageJ through the following procedure: the cross-sectional area of each sphere (*A_i_*) was first measured, and then the equivalent diameter (*d_i_*) was calculated:

(10) $$d_{i}=2\sqrt{A_{i}/\pi }.$$

*A_i_* and *d_i_* were then used to calculate *d_avg_*. For this purpose, roughly 100 spherulites were considered per each sample.

## 3. Results and Discussion

### 3.1. Schematic of RGO Synthesis and Morphological Configuration of Unfilled and Filled Binary Systems

**Figure 1:**
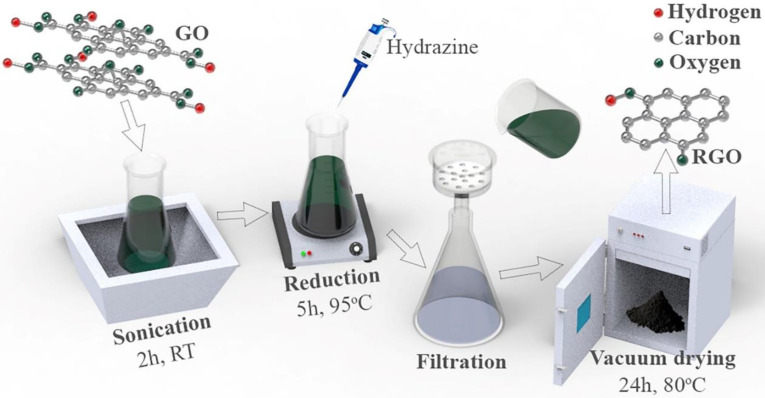
Schematic of RGO synthesis.

Figure 1 shows a schematic of RGO synthesis via GO reduction (explained step by step in Section 2.2).

GO has several oxygen-related functionalities, being hydroxyl, carboxyl, epoxy, and carbonyl moieties [23]. Upon reduction with hydrazine, most of these functional groups were removed from the main lattice, almost fully recovering the pure graphene structure. Indeed, as can be seen from Figure 1, RGO had very few oxygen-containing moieties, compared to GO, with the sp^2^ graphitic structure being mostly restored. This is supported by the experimental findings reported in the Supplementary Materials of our recent study [24]. In addition, GO reduction led to a co-factor (RGO) that had an improved exfoliation degree (fewer stacked layers), reduced interlayer distance, and increased aspect ratio and surface area, as shown in other research studies [25]. 

**Figure 2:**
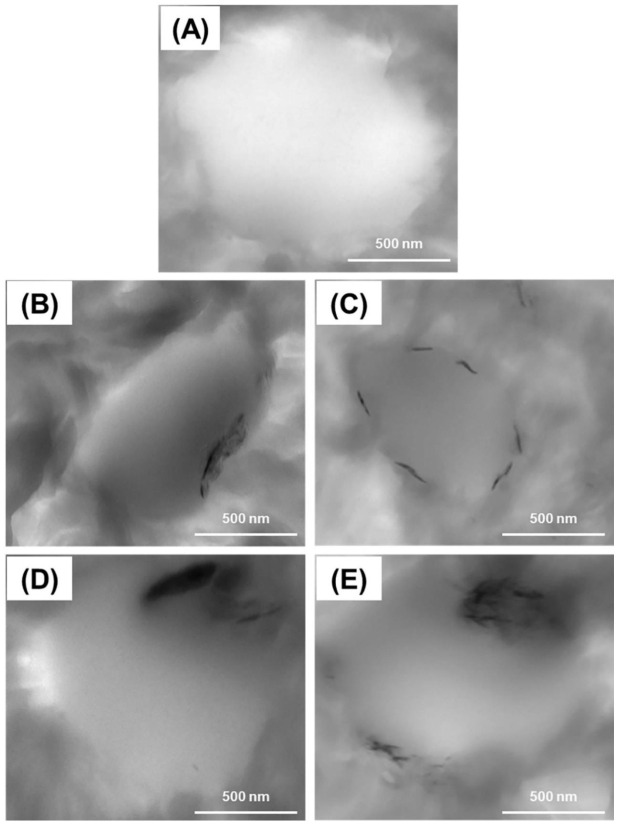
TEM images of PE90 (**A**), PE90GO2 (**B**), PE90RGO2 (**C**), PE90GO4 (**D**), and PE90RGO4 (**E**).

TEM micrographs of PE90, PE90GO2, PE90RGO2, PE90GO4, and PE90RGO4 are illustrated in Figure 2, with the intent of investigating GO and RGO exfoliation degree and dispersion state (PE phase, PP phase, or PE/PP interface).

In all the images, a PP droplet can be seen roughly in the center (smooth area), whereas the major phase (PE) is the rough area around. For filled binary systems, the co-factor was always localized at the PE/PP interface. This was the expected outcome of our strategic mixing of the three components. Nevertheless, RGO had a greater exfoliation degree and was better distributed at the blend interface (Figure 2C,E) compared to GO (Figure 2B,D), because the co-factor obtained upon chemical reduction (RGO) was better exfoliated and had less stacked layers than GO. When comparing RGO2 with RGO4, the latter had an agglomerated structure (Figure 2E) compared to the former (Figure 2C), since there was a restacking of graphitic platelets upon increasing the filler amount, due to Van der Waals attractions between the layers [26]. Consequently, compared to PE90RGO4 (Figure 2E), the most homogeneous distribution of the co-factor at the PE/PP interface was observed in the case of PE90RGO2 (Figure 2C). This also led to the most uniform minor phase dispersion into the major phase, with the PP size being the smallest, as indicated by Scanning Electron Microscopy (SEM) studies in our previous work [24]. Therefore, since morphology is usually correlated with properties, PE90RGO2 should have the greatest performance. Thermal properties characterization (Section 3.2) validated this hypothesis.

**Figure 3:**
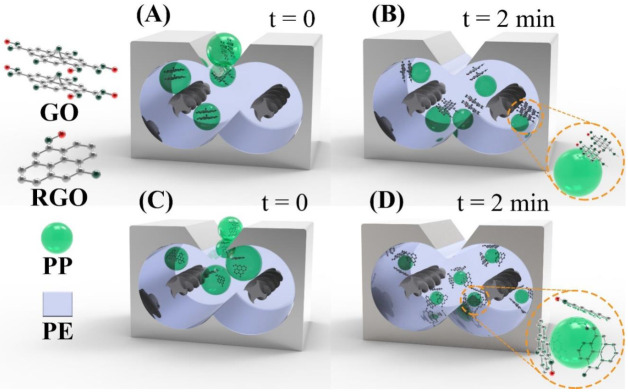
Mechanistic representation of morphological configuration of PE/PP/GO (**A** and **B**) and PE/PP/RGO (**C** and **D**) during step two of the ternary system synthesis.

Figure 3 graphically explains step two of the manufacturing process of PE/PP/GO (Figure 3A,B) and PE/PP/RGO (Figure 3C,D). The denotations t = 0 and t = 2 min represent the beginning and end of the mixing between the PP/Filler and PE, respectively.

As can be seen from Figure 3B,D, the sequential mixing procedure induced GO and RGO to be selectively localized at the biphasic system interface. This was thermodynamically explained in our previous research work [24], and a brief description is given as follows. The surface energy (SE) values of PE, PP, GO, and RGO were 38.4, 34.2, 132.3, and 115.3 mJ/m^2^, respectively. Since GO and RGO had SE values closer to that of PE than that of PP, the co-factor preferred to be dispersed into the major phase. Thus, when employing step two of the ternary system manufacturing procedure (PP/Filler mixed with PE), the co-factor migrated from the micro phase to the macro phase. At the same time, the mixing speed and time were controlled appropriately to induce the filler to stop its migration in time to localize itself at the PE/PP interface. Nevertheless, because of the presence of many oxygen-related functionalities on its structure, GO has a multilayered and agglomerated configuration [27]. Consequently, it was not homogeneously distributed at the PE/PP interface (Figure 3B, as well as Figure 2B,D), and PP was not uniformly dispersed in PE, as confirmed by SEM analysis in our recent work [24]. Therefore, the PE/PP/GO properties were barely enhanced. On the other hand, RGO was better exfoliated, with a higher aspect ratio, thus being more homogeneously distributed at the PE/PP interface (Figure 3D, as well as Figure 2C,E) with respect to GO. This led to a much finer PE/PP/RGO morphology, with PP being smaller in size and having a more uniform droplet diameter distribution, compared to what was seen for PE/PP/GO [24]. Consequently, a significant increase in PE/PP/RGO performance was registered (specifically thermal properties), as shown in Section 3.2.

### 3.2. Thermal Properties of Unfilled and Filled Binary Systems

**Figure 4:**
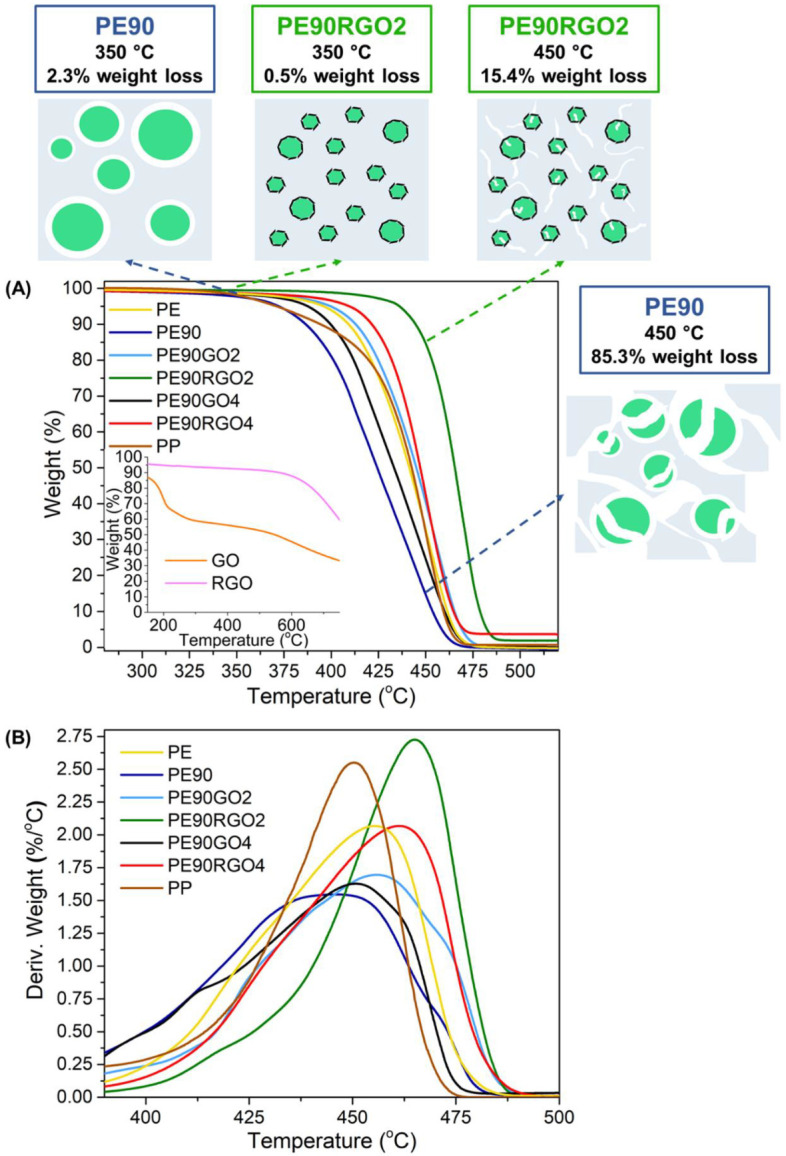
Weight over temperature (**A**) and derivative of weight over temperature (**B**) for unfilled and filled binary systems. (The insert graph of Figure 4A depicts weight over temperature for pure GO and RGO).

Figure 4A illustrates the TGA curves (at a rate of 10 °C/min) of unfilled and filled binary systems, whereas the insert graph represents the TGA curves of pure GO and RGO. Figure 4B depicts the weight derivative over temperature for unfilled and filled binary systems. Table 2 indicates Td_99_, the temperature at which thermal degradation begins, Td_50_, the temperature referring to 50% weight loss, and Td_10_, the temperature indicating 90% weight loss. These temperatures could be obtained from the main TGA graph. Table 2 also shows Td_max_ (temperature of maximum degradation), which could be measured from the insert TGA graph. 

Table 2 also depicts the HDT values for unfilled and filled blends. 

**Figure 5:**
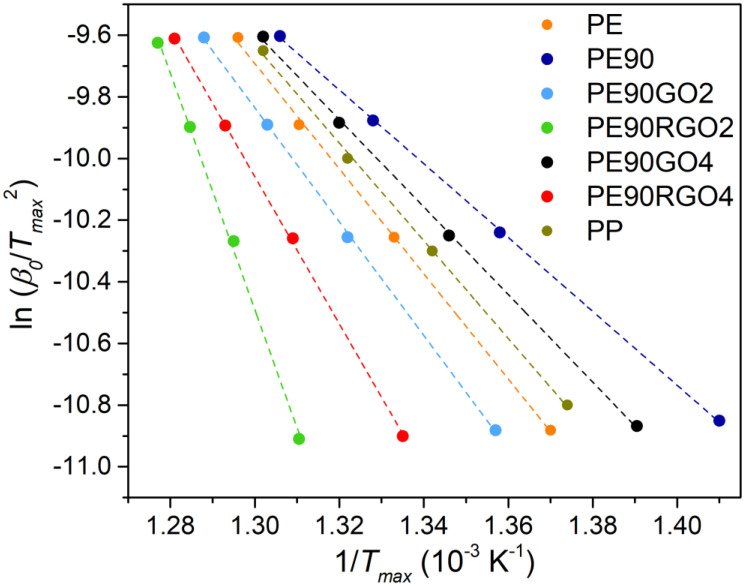
Kissinger plots for activation energy of thermal degradation of unfilled and filled binary systems.

Furthermore, the quantification of the material’s thermal stability was performed by calculating E_T_ (Table 2). For this purpose, four different heating rates were employed, as explained in Section 2.4. Figure 5 shows the plots of “ln(*β*_0_/*T_max_*^2^)” versus “1/*T_max_*”, and the activation energy value was taken from the slope. 

Compared to the neat binary system (PE90), Td_99_, Td_50_, Td_10_, Td_max_, E_T_, and HDT were higher for filled biphasic systems because of the co-factor gas barrier activity, which hindered the gaseous molecules’ diffusion coming from the two polymers [28]. Consequently, the thermal decomposition was delayed, and the thermal stability was increased. The co-factor, placed at the blend interface, also improved the PE/PP interfacial adhesion, thus enhancing the material’s heat deformation resistance [29]. A higher boost in thermal properties was registered when introducing RGO as a filler, instead of GO, as the more uniformly distributed co-factor not only led to stronger interactions between the two polymeric phases but also formed stronger charred layers on the polymers’ surfaces, thus more efficiently disrupting the volatile degraded matter [30]. Moreover, RGO4 had a lower impact on the material’s thermal performance compared to RGO2, because the former was more agglomerated (Figure 2E) than the latter (Figure 2C), hence not bringing high PE/PP interfacial adhesion and not preventing the emission of gaseous molecules during the decomposition of the two polymers as effectively as RGO2. Another outcome of the performed TGA study was that above 500 °C, the residual (non-degraded) PE90RGO2 sample was 2 wt %, while the non-degraded PE90RGO4 was about 4 wt %. These quantities represented the co-factor weight content in the two ternary systems. This is demonstrated by the insert graph in Figure 3A. Since RGO begins to degrade only after 600 °C, the samples PE90RGO2 and PE90RGO4 still contain the full amount of RGO in them at a temperature of about 500 °C. The insert graph in Figure 3A also demonstrates that the thermal stability of pure GO and RGO is higher than that of the polymeric ternary systems, especially at high temperatures. Moreover, Figure 4A shows that PE90 lost 2.3% of its total weight at 350 °C, while 85.3% was lost at 450 °C. On the other hand, the PE90RGO2 weight loss was 0.5% at 350 °C and 15.4% at 450 °C. Therefore, in the temperature range of 100 °C, a huge difference in weight loss was observed for PE90, whereas a less significant weight loss occurred for PE90RGO2. The reason why this happened is graphically explained by the 2D drawings in Figure 4. PE90 is comprised of two immiscible polymers (PE and PP); thus, its weakest point is the PE/PP interface. This means that at high temperatures (for example 450 °C), a high degree of material degradation is expected to take place at the blend interface, followed by the propagation of the cracks through the macro phase (PE) and the micro phase (PP), causing larger cracks. Conversely, in the PE90RGO2 system, RGO was almost homogeneously distributed at the blend interface. This increased the interfacial adhesion between the two polymer phases, thus making the PE/PP interface stable. As a result, a less severe thermal degradation should be observed, with much smaller fractures occurring in the PE and PP phases, even at high temperatures, e.g., 450 °C, therefore leading to a more thermally stable material. Lastly, Figure 4 and Figure 5, as well as Table 2, indicate that the TGA temperatures and E_T_ of PE90RGO2 were higher than those of virgin PP, while the HDT value of PP was not too far from that of PE90RGO2. Our previous work showed that PE90RGO2 was better performant than neat PP in the case of mechanical properties, too (strength, stiffness, and toughness) [24]. This proved that the proposed GO chemical reduction and sequential mixing of the three components yielded a highly performant PE-rich product that has the potential to replace more expensive PP-based ones in energy and automotive industries for today’s highly demanding applications.

### 3.3. PE Spherulitic Morphology

**Figure 6:**
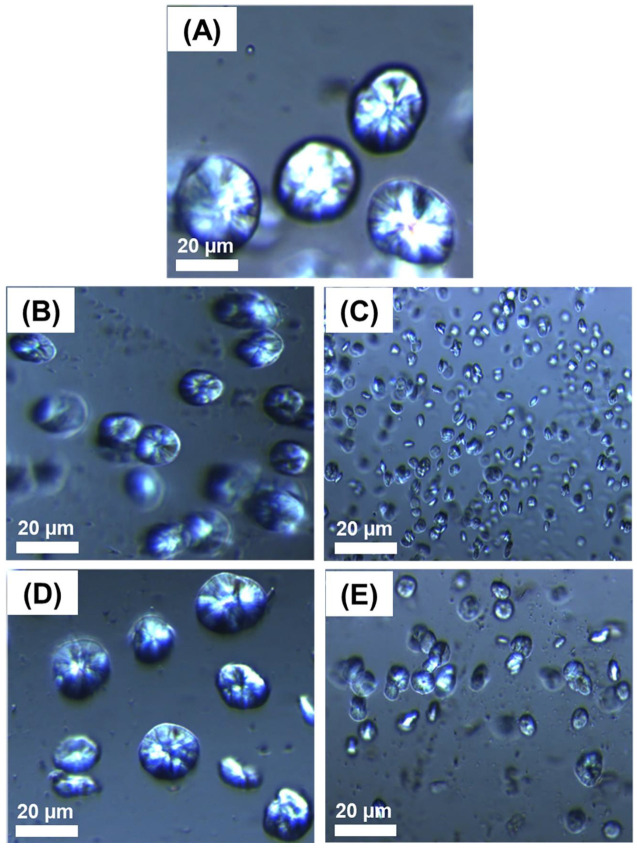
Polarized Light Optical Microscopy (PLOM) micrographs of PE crystal growth in PE90 (**A**), PE90GO2 (**B**), PE90RGO2 (**C**), PE90GO4 (**D**), and PE90RGO4 (**E**).

Figure 6 represents PLOM images of PE crystal growth in the different systems considered. In the case of a neat binary system (Figure 6A), only few big PE crystals are seen. Since the two polymers are immiscible, PP has no ability to influence PE spherulite growth. Conversely, once the co-factor was added (Figure 6B–E) and localized at the binary system interface (Figure 2B–E), thanks to the employed strategic mixing of the three components, droplet coalescence was hindered, and a finer dispersion of the minor phase into the major phase was registered, as confirmed by the SEM analysis outlined in our previous work [24]. Consequently, PP provided more nucleating sites for PE crystallization, increasing the spherulitic nucleation density [31].

As can been seen from Figure 6C, the best nucleating process for PE crystallization is constituted by the synergistic addition of PP and RGO2. Indeed, among all the systems under investigation, PE90RGO2 was the one in which the highest nucleation density of PE was observed, with its spherulites being the smallest, as well as the most uniform in size. In addition, the calculated average diameter of PE crystals in the PE90RGO2 system was 2.2 (±0.1) µm, while it was 28.8 (±0.3), 13.6 (±0.2), 18.9 (±0.3), and 6.7 (±0.2) µm in the configurations PE90, PE90GO2, PE90GO4, and PE90RGO4, respectively. The values in parentheses represent the standard deviation. Furthermore, PE had the fastest and easiest crystallization in the PE90RGO2 system, because RGO2 was highly exfoliated and homogeneously distributed at the PE/PP interfacial area (Figure 2E). This promoted a much more uniform minor phase dispersion into the major phase compared to the other ternary systems investigated (proven, in our previous work [24], by SEM studies), hence increasing the number of heterogeneous sites for PE crystals to grow. It is also possible that the RGO2-saturated interfacial area between the two polymeric phases served as an extra heterogeneity for the nucleation of PE [32].

### 3.4. PE Thermal and Crystallization Behavior in Unfilled and Filled Binary Systems

**Figure 7:**
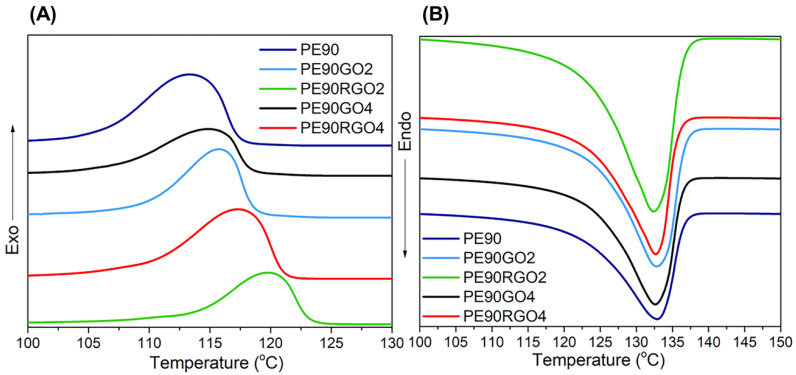
Cooling (**A**) and heating (**B**) thermograms of PE in unfilled and filled binary systems.

Figure 7 depicts cooling and heating thermograms of PE in unfilled and filled biphasic systems at cooling and heating rates of 10 °C/min.

T_c_, T_m_, X_c_, t_c_, and supercooling (ΔT = T_m_ − T_c_) could be determined from the cooling thermogram (Figure 7A), and they are represented in Table 3. 

**Figure 8:**
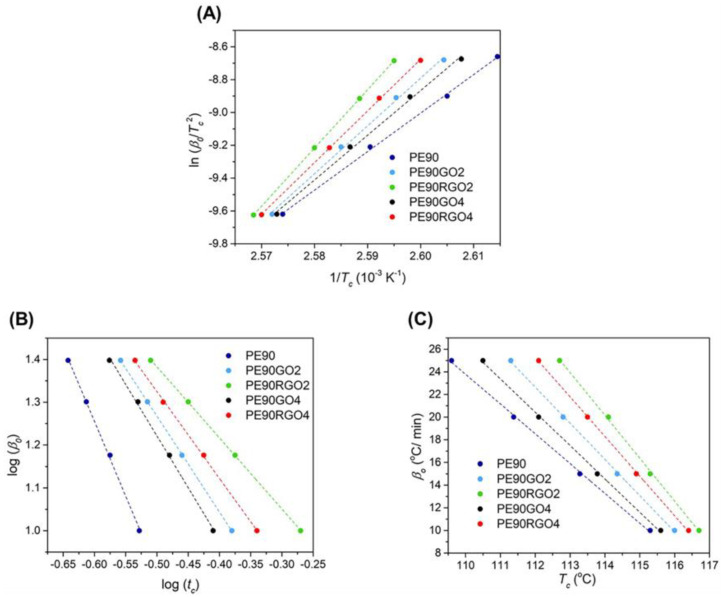
Kissinger (**A**), Liu (**B**), and Khanna (**C**) plots for PE non-isothermal crystallization in unfilled and filled binary systems.

According to the literature, an increase in T_c_ and a decrease in t_c_ indicate nucleation-controlled polymer crystallization [33]. Furthermore, when a polymer crystallizes with less supercooling, it crystallizes more perfectly [34]. Table 3 shows that PE90RGO2 was characterized by the highest T_c_ and X_c_, as well as the lowest t_c_. Moreover, even though T_m_ hardly changed for all the samples under consideration, the minimum value of ΔT was reached in the case of PE90RGO2. Therefore, the best nucleating effect on PE crystallization was achieved when the micro phase PP was paired with the co-factor RGO2, since the latter was highly exfoliated and selectively localized at the binary system interface, bringing a high surface area of interaction with the two polymers, hence increasing their synergy. This is also validated by other works [35,36] available in the literature, which study the crystallization of PE in nanocomposites by means of Small Angle X-Ray Scattering (SAXS), Wide Angle X-Ray Scattering (WAXS), and X-Ray Diffraction (XRD). Figure 8 indicates the “ln(*β*_0_/T_c_^2^)” versus “1/T_c_” plot (Kissinger), the “log *β*_0_” versus “log t_c_” plot (Liu), and the “*β*_0_” versus “T_c_” plot (Khanna) for PE non-isothermal crystallization in the systems investigated.

In all the cases, each plot was generated by performing DSC analysis at four different cooling rates, as explained in Section 2.6. The values of *E_c_*, *F*(*T*), and *CRC* are depicted in Table 4. 

Research studies indicate that a faster crystallization occurs at lower values of *E_c_* [37,38] and *F*(*T*) [39], as well as higher values of *CRC* [40]. Table 4 shows that when the nucleating agent was represented by the pair PP + filler (samples PE90GO2, PE90RGO2, PE90GO4, PE90RGO4), compared to only PP (sample PE90), the *E_c_* and *F*(*T*) of PE were always lower, while the *CRC* of PE was always higher. This was because the employed sequential mixing induced the co-factor to be selectively localized at the biphasic system interface (Figure 2B–E), increasing its interaction with the two polymers [41] and hence improving the nucleating effect of PP on PE crystallization. Moreover, since the *E_c_* and *F*(*T*) of PE were lower and *CRC* was higher, when the filler was RGO, as opposed to GO, the PP nucleating activity on PE crystallization was more effective. This happened because the co-factor was better exfoliated and covered more interfacial area (Figure 2C); hence, it had a higher surface area of interaction with the two polymeric phases. When comparing different amounts of RGO, *E_c_* and *F*(*T*) of PE reached the lowest value, while *CRC* reached the highest value when the co-factor was RGO2 instead of RGO4, demonstrating that the best nucleating effect of PP + filler on PE crystallization was reached when RGO was introduced with a 2% weight concentration, rather than 4%. Consequently, in the PE90RGO2 configuration, a very uniform size distribution of PE crystals was registered, as confirmed by PLOM studies in Section 3.3. This is also validated by other works [35,36] available in the literature that study the crystallization of PE in nanocomposites, by means of SAXS, WAXS, and XRD. Overall, by pairing the outcome of studies on PE non-isothermal crystallization kinetics with those of optical microscopy, it can be claimed that in the system PE90RGO2, among all the polymeric systems investigated, PE crystallization was not only the fastest (lowest E_C_ and F(T), as well as highest CRC), but it was also characterized by the most uniform spherulite size (Figure 6C). Moreover, the experimental findings (TGA, HDT, DSC, and PLOM) agreed with the theoretical studies (crystallization kinetics), demonstrating that an improved PE non-isothermal crystallization led to a greater increase in thermal stability and heat deformation resistance for the PE-rich PE/PP/RGO ternary system.

## 4. Conclusions

GO was modified to obtain RGO, which was then thermodynamically localized at the interface of a PE-based PE/PP biphasic polyolefin system. The impact of the produced RGO on ternary system’s thermal stability and heat deformation resistance was investigated. RGO-filled biphasic polyolefin systems had greater performance than those of PE/PP/GO, as GO modification brought a better exfoliated co-factor, which covered the binary system interfacial area more evenly (confirmed by TEM), leading to a finer dispersion of PP into the major phase. This translated into stronger interactions between the co-factor and the two polymers, thus hindering their chains’ mobility. Consequently, thermal degradation and heat deformation were delayed. In terms of different RGO loads, a stronger impact on the ternary system’s performance enhancement was registered when the filler weight fraction was 2% as opposed to 4%, because RGO2 had a less agglomerated configuration than RGO4. Therefore, the co-factor was more homogeneously distributed at the PE/PP interface (confirmed by TEM) and the micro phase was better dispersed into the macro phase, leading to greater synergy between the two polymers. This was also confirmed by the activation energy of thermal degradation, which was the highest for PE90RGO2. Another interesting feature of this research work was the investigation of PE non-isothermal crystallization in unfilled and filled binary systems. RGO and PP played a synergistic nucleating effect on PE crystallization, making it faster and easier, with spherulites having a more uniform size distribution (confirmed by PLOM). The most improved PE crystallization was registered when the filler was RGO2, with *E_c_*, *t_c_*, Δ*T*, and *F*(*T*) being the lowest, and *T_c_*, *X_c_*, and *CRC* being the highest. Lastly, the TGA properties of PE90RGO2 were higher than those of neat PP, while their HDT values were found to be close to each other. This generates potential for industries, such as the energy and automotive sectors, to replace PP-rich products with those based on a more abundant, cheaper, yet performant PE, thus satisfying the strict requirements of emerging engineering applications.

## Figures and Tables

**Table 1:** Sample denotations and weight fractions. GO: Graphene Oxide, HDPE: High-Density Polyethylene, iPP: Isotactic Polypropylene, PE: Polyethylene, RGO: Reduced Graphene Oxide.

Denotations	HDPE (wt %)	iPP (wt %)	GO (wt %)	RGO (wt %)
**PE90**	90	10	0	0
**PE90GO2**	88.2	9.8	2	0
**PE90RGO2**	88.2	9.8	0	2
**PE90GO4**	86.4	9.6	4	0
**PE90RGO4**	86.4	9.6	0	4

**Table 2:** Thermal properties of unfilled and filled binary systems. HDT: Heat Deformation Temperature.

	Td_99_ (°C)	Td_50_ (°C)	Td_10_ (°C)	Td_max_ (°C)	E_T_ (KJ/mol)	HDT (°C)
**PE**	340 (±1.6)	442 (±1.4)	463 (±1.4)	455 (±1.5)	159.4 (±0.6)	48.6 (±0.5)
**PE90**	301 (±1.4)	422 (±1.7)	456 (±1.1)	443 (±1.2)	124.2 (±0.9)	47.2 (±0.4)
**PE90GO2**	334 (±1.1)	451 (±1.3)	465 (±1.2)	457 (±1.4)	162.1 (±1.1)	57.3 (±0.5)
**PE90RGO2**	359 (±1.2)	462 (±1.2)	487 (±1.3)	467 (±1.1)	238.7 (±0.7)	66.7 (±0.3)
**PE90GO4**	326 (±1.2)	435 (±1.1)	456 (±1.2)	451 (±1.4)	153.6 (±0.8)	52.4 (±0.4)
**PE90RGO4**	335 (±1.4)	456 (±1.1)	466 (±1.1)	462 (±1.3)	196.8 (±0.6)	59.9 (±0.4)
**PP**	339 (±1.3)	441 (±1.2)	457 (±1.4)	450 (±1.3)	157.1 (±0.8)	70.8 (±0.3)

**Table 3:** Crystallization and thermal parameters of PE in unfilled and filled binary systems.

	T_c_ (°C)	T_m_ (°C)	ΔT (°C)	t_c_ (min)	X_c_ (%)
**PE90**	113.4 (±1.4)	132.6 (±0.6)	19.2 (±0.6)	0.53 (±0.04)	62.1 (±0.8)
**PE90GO2**	116.3 (±1.1)	132.4 (±0.6)	16.1 (±0.4)	0.40 (±0.01)	64.8 (±0.9)
**PE90RGO2**	120.6 (±2.6)	132.2 (±0.8)	11.6 (±0.4)	0.29 (±0.06)	74.3 (±1.1)
**PE90GO4**	114.9 (±1.1)	132.5 (±0.5)	17.6 (±0.5)	0.45 (±0.02)	62.9 (±0.9)
**PE90RGO4**	117.8 (±1.2)	132.3 (±0.7)	14.5 (±0.5)	0.38 (±0.02)	66.3 (±0.7)

**Table 4:** Kissinger, Liu, and Khanna crystallization parameters of PE in unfilled and filled binary systems. CRC: crystallization rate coefficient.

	*E_c_* (KJ/mol)	*F*(*T*)	*CRC* (min^−1^)
**PE90**	283.5 (±1.2)	4.4 (±0.03)	2.97 (±0.04)
**PE90GO2**	269.8 (±0.9)	2.6 (±0.02)	3.25 (±0.03)
**PE90RGO2**	201.7 (±1.1)	1.3 (±0.02)	3.74 (±0.04)
**PE90GO4**	276.4 (±1.2)	3.1 (±0.04)	3.12 (±0.02)
**PE90RGO4**	216.1 (±0.8)	2.2 (±0.03)	3.61 (±0.02)
